# Monoamine Oxidase (MAO) as a Potential Target for Anticancer Drug Design and Development

**DOI:** 10.3390/molecules26196019

**Published:** 2021-10-04

**Authors:** Reem Aljanabi, Lina Alsous, Dima A. Sabbah, Halise Inci Gul, Mustafa Gul, Sanaa K. Bardaweel

**Affiliations:** 1 Department of Pharmaceutical Sciences, School of Pharmacy, University of Jordan, Amman 11942, Jordan; aljanabireem@live.com (R.A.); linuciasous@gmail.com (L.A.); 2 Department of Pharmacy, Faculty of Pharmacy, Al-Zaytoonah University of Jordan, P.O. Box 130, Amman 11733, Jordan; dima.sabbah@zuj.edu.jo; 3 Department of Pharmaceutical Chemistry, Faculty of Pharmacy, Ataturk University, Yakutiye 25030, Turkey; incigul1967@yahoo.com; 4 Department of Physiology, School of Medicine, Ataturk University, Yakutiye 25030, Turkey; mustafagul@hotmail.com

**Keywords:** monoamine oxidase, cancer, metastasis, inhibitors, QSAR, pharmacophore

## Abstract

Monoamine oxidases (MAOs) are oxidative enzymes that catalyze the conversion of biogenic amines into their corresponding aldehydes and ketones through oxidative deamination. Owing to the crucial role of MAOs in maintaining functional levels of neurotransmitters, the implications of its distorted activity have been associated with numerous neurological diseases. Recently, an unanticipated role of MAOs in tumor progression and metastasis has been reported. The chemical inhibition of MAOs might be a valuable therapeutic approach for cancer treatment. In this review, we reported computational approaches exploited in the design and development of selective MAO inhibitors accompanied by their biological activities. Additionally, we generated a pharmacophore model for MAO-A active inhibitors to identify the structural motifs to invoke an activity.

## 1. Introduction

By 1928, Mary Bernheim discovered the first enzyme of monoamine oxidase, and it was called tyramine oxidases [1]. Monoamine oxidases are in the flavin protein family, which is essentially composed of flavin amine oxidoreductases [2]. Monoamine oxidase can be classified into two types: monoamine oxidase A (MAO-A) and monoamine oxidase B (MAO-B). MAO-A is present in the gastrointestinal tract, lung, liver, and placenta, whereas MAO-B is present in blood platelets [3]. Monoamine oxidase A (MAO-A), a mitochondrial oxidative enzyme in the broad class of deaminating oxidases [4], essentially catalyzes the conversion of biogenic amines, such as dopamine and epinephrine, into their corresponding aldehydes through oxidative deamination with the concurrent production of reactive oxygen species (ROS) [5,6,7]. The prevalent existence of MAO-A is accredited to a conserved biological role in amine metabolism [5], and therefore it may be engaged in crucial cellular functions, such as monitoring cell growth and differentiation, maintaining the polyamine reservoir, and regulating levels of neurotransmitters [8].

Due to the essential role of MAO-A in preserving functional levels of neurotransmitters, the implications of its abnormal activity have been linked to several neurological disorders. For instance, increased activity of MAO-A has been associated with depression and anxiety [9,10]. On the other hand, deficiency of MAO-A enzymatic activity has been demonstrated in patients with mental retardation and abnormal behavior [11]. Moreover, the pathogenesis of neurodegenerative diseases, such as Parkinson’s and Alzheimer’s diseases, was proposed to be mediated by increased expression and activity of MAO-A [12,13]. Notably, several cardiovascular diseases, including heart failure [14], vascular remodeling [15] and myocardial injury [16], have been also related to abnormal levels and activity of MAO-A.

Monoamine oxidase B can be classified as a flavin adenine dinucleotide (FAD)-dependent mitochondrial enzyme. It acts primarily by catalyzing the oxidative deamination of different amines [17]. MAO-B shows preferential selectivity toward two substrates, which are 2-phenylethylamine and benzylamine. Dopamine and tyramine are also considered substrates for MAO-B. Selegiline (L-deprenyl), one of the first selective MAO-B inhibitors with an enhanced profile, has been used for almost 40 years to treat Parkinson’s disease. Glial brain cells are rich in MAO-B enzyme, especially in the neighborhood of dopaminergic synapses [18]. MAO-B participates mainly in storage regulation, release, and concentrations of biogenic amines in the synaptic cleft. Reactive oxygen species (ROS) are generated through monoamine substrate oxidation via MAO-B. Oxidation overabundance encourages neurotoxins synthesis. Tetrahyroisoquinolone (TIQ) and 6-hydroxydpamine are two of the most common neurotoxins whose high concentrations fasten chronic neurodegenerative disease [19].

During the last decade, several reports were published connecting the MAO-A-mediated production of ROS with tumor development and progression. Patients with advanced prostate cancer (PCa) showed elevated expression of MAO-A [20,21]. Oxidative stress and the resulting DNA damage caused by ROS were used to explain tumor initiation and progress in several types of cancers [22,23]. In addition, the ability of cancer cells to migrate from their primary location to another tissue appears to be linked to signaling pathways that involve MAO-A activity [24]. Interestingly, several MAO-A inhibitors were reported to modulate cell proliferation and result in cell cycle arrest in a dose-dependent trend. Cancer cell death induced by apoptosis pathways has been reported to be the main effect of certain MAO-A inhibitors on prostate cancer cells [25]. It is not only synthetic drugs that demonstrate anticancer activity; curcumin, a bioactive phytochemical compound that is proposed to work through the inhibition of MAO-A/mTOR/HIF-1α signaling pathways, revealed a reduction in cancer-associated fibroblast-induced invasion and ROS production in prostate cancer [26,27,28]. From this perspective, we review the existing studies on the role of MAO-A and MAO-B in cancer development and progression with a special focus on the design and use of MAO-A inhibitors in cancer chemotherapy.

## 2. MAO-A’s Location and Function

**Figure 1:**
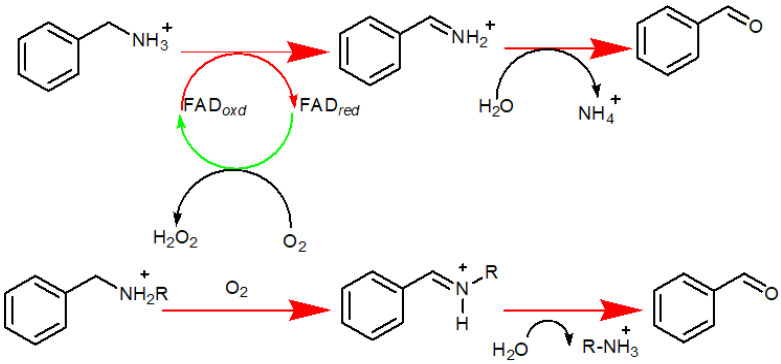
Oxidation of primary and secondary amines into their respective imine form, followed by nonenzymatic hydrolysis to their corresponding aldehydes or ketones.

MAO-A gene is an X-linked gene, located on the X chromosome (Xp11.23), encoding the outer mitochondrial membrane MAO-A protein [29]. The protein is a pro-oxidative enzyme that is extensively present in all the mammalian cell types except erythrocytes [4]. It catalyzes the oxidation of primary and secondary amines into their respective imine form; followed by nonenzymatic hydrolysis to the corresponding aldehydes or ketones [30], as illustrated in Figure 1. The enzyme has a covalently bound FAD cofactor linked via a thioether bridge [31]. MAO-A favorably catalyzes the metabolism of serotonin (5-hydroxytryptamine), norepinephrine, and dopamine [31,32]. MAO-A plays a key role in many neuropsychiatric diseases, as it is involved in controlling levels of neurotransmitters [33]. In addition, MAO-A contributes to ROS generation through its catalytic by-product hydrogen peroxide (H_2_O_2_) [34]. Reactive oxygen species levels control normal mitochondrial functions and may result in multiple dysfunctions as well [35].

## 3. MAO-A’s Role in Cancer

The MAO-A-mediated production of ROS could lead to DNA damage and oxidative injury of cells and thus may participate in tumor initiation and progression. Many studies have shown that MAO-A overexpression is associated with an increased risk of cancer [23,36]. Aggressive prostate cancer (PCa) demonstrated high expression of MAO-A [21]. Hodorova et al. reported that renal cell carcinoma may have high-grade MAO-A expression [37]. It has also been proposed that MAO-A expression is relatively increased in human glioma tissues and cell lines. The chemical inhibition of MAO-A with clorgyline, a selective and irreversible inhibitor of MAO-A, was effectively cytotoxic for glioma and decreased the invasion in vitro [38,39].

MAO-A protein and mRNA expression were significantly higher in non-small cell lung carcinoma (NSCLC) tissues compared to the matched non-tumor adjacent lung tissues [40]. Moreover, MAO-A expression was linked to the clinical stage and lymph node metastases [41]. Recently, it has been suggested that MAO-A may have a role in promoting the progression of NSCLC by regulating the epithelial to mesenchymal transition (EMT) process, a key step in cancer invasion and metastasis, by negatively affecting E-cadherin expression and positively affecting the expressions of N-cadherin [42,43,44].

One of the deadliest diseases affecting women is breast cancer [45]. Cancer progression, angiogenesis, and metastasis require more exploration, especially at the molecular level [46]. MAO-A appears to play a different role in breast cancer pathogenesis [47]. Based on the available literature, MAO-A was found at a low expression level in many types of breast cancers [48]. Interleukin-6 is a cytokine principally abundant in a number of inflammatory conditions [49]. Many reports proposed that cytokine is involved in cancer progression, metastasis, chemo-resistance, angiogenesis, and epithelial to mesenchymal transition [50]. Interleukin 6 (IL-6) is connected to more aggressive and invasive types of cancer. In addition, IL-6 acts primarily through activating a series of downstream signaling cascades, including GP130, JAK/STAT, MAPK, and AKT, which are all involved in cancer initiation and progression [51]. Bharti et al. reported that a low level of MAO-A promotes tumor angiogenesis and invasion in breast cancer in a hypoxic environment. IL-6/IL-6R was found to exert a negative regulation pattern on MAO-A activity [47]. Diacerein (Dia) acts through the inhibition of the IL-6/IL-6R signaling pathway, suppressing angiogenesis and invasion by up-regulating MAO-A expression [52].

## 4. MAO-B’s Role in Cancer

A few years ago, interest in MAO-B increased, as it was linked with a direct relationship to many types of cancer. A considerable number of studies showed that both MAOs have high levels in different cancer types [53]. Colorectal cancer (CRC) is considered one of the most common cancers worldwide, especially in Asia. Surgery is considered the first choice of treatment in most colorectal cancer cases. However, the rate of reoccurrence is about 30% due to distant metastasis, particularly in late-stage patients [54]. Yang et al. [55] studied MAO expression in colorectal cancer using in silico analysis and tissue microarrays. In 203 cases of colorectal adenocarcinoma, MAO-B demonstrated high expression in cancer tissues in comparison to normal tissues. The study compared MAO-B expression with clinicopathological parameters of patients. The results showed that high MAO-B expression in tissues related well with high reoccurrence rate and poor prognosis. On the other hand, MAO-B expression had a positive correlation with epithelial-to-mesenchymal-transition-related gene expression in CRC tissues [55].

One of the heterogeneous tumors is breast cancer. In 2020, breast cancer was classified as the world’s largest occurrence cancer [56]. Usually, breast cancer is categorized based on the expressed hormone receptors (estrogen, progesterone, human epidermal growth factor receptor 2), and it is divided into different subtypes (luminal A, luminal B, HER-2 type, triple negative breast cancer (TNBC)) [57]. As MAO-A showed high expression in luminal A and luminal B, MAO-B was highly expressed in TNBC with a p-value of 0.02. In contrast, a study reported that cells expressing estrogen-related receptor (ERR) showed high MAO-B expression as well [58].

Lung cancer is the leading cause of cancer death, constituting 25% of all cancer deaths [59]. One of the major problems challenging lung cancer treatment is the high level of ionizing radiation resistance that decreases radiation therapy effectiveness [60]. Ionizing radiation resistance is mainly attributed to nuclear factor kappa-light-chain-enhancer of activated B cell (NF-KB) pathway activation. One of MAO-B’s catalyzing products is hydrogen peroxide, which is important for the NF-KB activation pathway in NSCLC. It has been found that MAO-B is overexpressed in lung cancer cells in comparison to normal cells. MAO-B expression increased (mRNA and protein levels) in A549 and H1299 upon ionizing radiation (IR) treatment in a dose-dependent manner [61]. Therefore, it is concluded that MAO-B can be considered a biomarker for NSCLC and IR resistance. MAO-B acts primarily through NF-KB activation [62]. Danshensu is a traditional oriental medicine that has been shown to reduce IR resistance mainly through NF-KB activation. Danshensu works on reducing MAO-B activity and regaining the radio-sensitization of NSCLC [63].

Oral squamous cell carcinoma (OSCC) is one of the most prevalent cancer types in south Asia. An in silico drug design and molecular docking study identified Galuteolin and Linarin as potential leads for oral squamous cell carcinoma (OSCC) treatment [64]. Both Galuteolin and Linarin inhibited AKt1 and AKt2 proteins, but not MAO-B, which showed a decreased expression in OSCC tissues. On the other hand, Diosmetin, Acacetin, and Epicatechin appear to inhibit MAO-B selectively, but not AKt1 and AKt2 proteins. Consequently, it was concluded that MAO-B inhibitors could be used for the treatment of cancer types other than OSCC [64].

Young Oh et al. [65] studied MAO-B as a potential biomarker for the early detection of OSCC. The study included 34 samples from healthy individuals and 33 samples from OSCC patients. Real-time PCR for six genes was performed, and mRNA levels were compared. MAO-B showed decreased expression in OSCC patients in comparison to healthy individuals. MAO-B expression could be used as an early diagnosis indicator for OSCC [65].

In addition, gliomas appear to have a strong correlation with MAO-B; MAO-B has a high expression level in this type of cancer, especially with high-grade tumors [66]. Moreover, MAO-B has a strong correlation with hypoxia-inducible factor 1 alpha (Hif-1α) expression. Therefore, MAO-B can be considered a hot target for the treatment of Gliomas [67].

The gastrointestinal tract (GIT) contains many neurotransmitters, and MAO-B is one of the major metabolizing enzymes for these neurotransmitters [68]. Quantitative real-time PCR and the Seahorse assay were used to study the MAO-B in GI cancers. Norepinephrine levels showed high levels in gastric cancer tissues. MAO-A and MAO-B appeared to be expressed in low levels. The high levels of norepinephrine and low MAO-B expression could be a good target for immune therapy [69].

Human carcinogens such as Betal Quid and Areca Nut are usually associated with a high incidence risk of oral malignant disorders [70]. Arecolin is an alkaloid usually metabolized by MAO proteins with a concurrent production of reactive oxygen species. Decreased expressions of MAO-A and MAO-B were demonstrated in such cancerous tissues in comparison to non-cancerous tissues [71].

MicroRNAs (miRNA) could act either as tumor suppressers or cancer-promoting factors [72]. The effect of miR-522 in endometrial carcinoma was studied, and it has been shown that miR-522 decreased MAO-B expression. This effect usually occurs due to miR-522 binding to MAO-B with a putative site. Therefore, miR-522 accelerated endometrial carcinoma through MAO-B inhibition [73].

## 5. Structural Design of MAO-A Inhibitors

**Figure 2:**
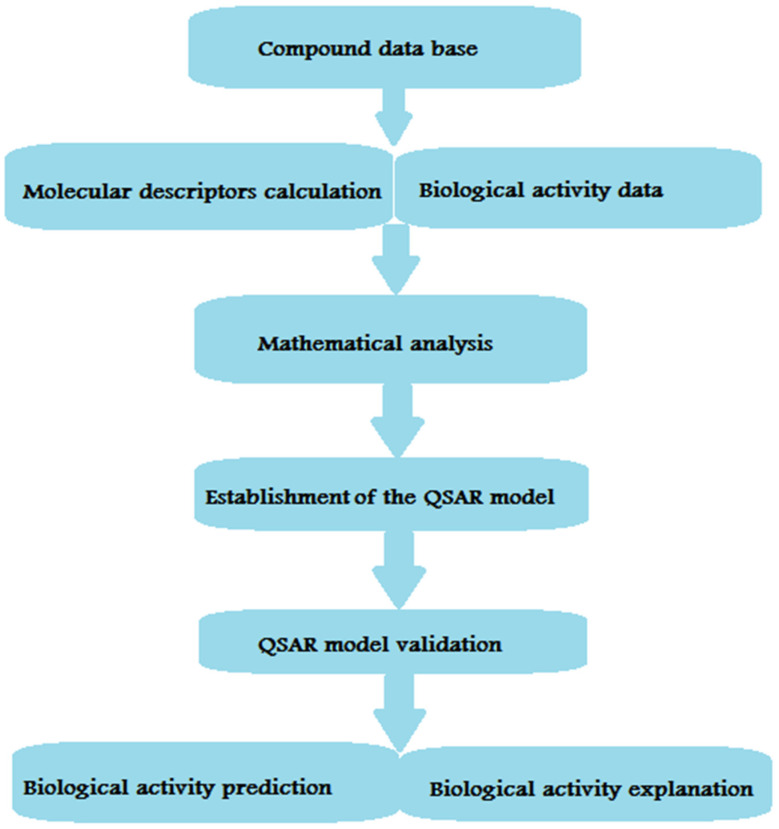
Main steps involved in the development of a QSAR model.

The crystal structures of the human MAO-B complex with isatin (PDB ID: 2BK5) [74] and the MAO-A complex with clorgyline (PDB ID: 2BXS) [75] were released in 2002 and 2005, respectively. Since MAO enzymes are involved in diverse biological pathways of clinical significance, they seem to be promising targets in pharmacological research [76,77]. Due to their potential clinical importance, rigorous research has been attempted to retrieve new compounds with MAO-suppressive activity with few adverse effects. One of the main adverse effects demonstrated by the first generation of irreversible inhibitors was liver toxicity or the ‘cheese effect’ distinguished by hypertensive crisis [78,79]. The release of two MAO crystal structures encouraged researchers in the same field to delineate the structural basis of ligand–MAO complex formation [80,81,82,83]. Such a finding is significant in the rationale design and development of novel MAO inhibitors. Ligand-based drug design approaches are successful in designing and optimizing new compounds with better activity, whereas structure-based drug design strategies explore ligand/MAO interaction and elucidate potential mechanisms of action [84]. Ligand-based approaches inspect molecular fingerprints (similar structural features have similar biological activities). Ligand-based tactics accommodate a quantitative structure-activity relationship (QSAR), having 2D and 3D physicochemical descriptors [85,86], 3D-comparative molecular field analysis (CoMFA) [87], 3D-pharmacophore [88], or ligand-centric network models [89]. QSAR studies are wide-spread ligand-based approaches in medicinal chemistry [90]. The major steps implicated in QSAR development are illustrated in Figure 2.

After the QSAR model is developed, the next step is predicting the biological activity of new compounds and interpreting the results to better understand the mechanism of action. Different methodologies have been developed to contrast the spread of 3D descriptors’ space as a 3D structure for ligand/receptor interaction. In order to generate electronic, steric fields, or pharmacophore modeling, an alignment of the given structure with the calculation of the 3D molecular conformations is required [91]. The developed pharmacophore modeling may explain the biological/chemical complementarity with the target. One of the common challenges that faces this type of modeling is the final model construction, especially when large structural differences exist in the compounds. However, pharmacophore modeling is still a reliable approach to explain how structurally different ligands interact with their targets [92]. Network analysis is another type of model that provides a pharmacological general strategy. One of the applications in drug design is computational biology network modeling that provides a tool to explain the relationships between ligands and pharmacological targets [93,94]. Improving efficiency in the process of drug design and discovery is mainly achieved through analysis of ligand–protein networks that present a better understanding of the relevance of biological targets. To endow insights into the relationship between MAO activity and structural scaffolds, a series of ligand-based models are illustrated below [95].

## 6. Xanthone Derivatives

**Figure 3:**
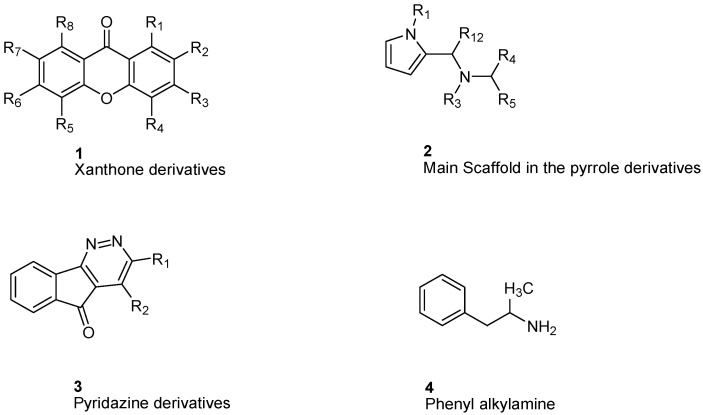
Main scaffolds of Xanthone, Pyrrole, Pyridazine, and Phenyl alkylamine derivatives studied as MAO-inhibitors (R_1_ = OH, R_2_ = MeO, R_3_ = MeO, R_4_ = H or OH R_5_ = H, R_6_ = CHMe_2_, R_7_ = NHMe, NMe_2_, NH_2_, R_8_ = H).

Gnerre et al. [96] studied MAO inhibitors in a set of 59 natural and synthetic xanthones derivatives (Figure 3). The compounds showed more selectivity toward MAO-A than MAO-B with IC_50_ values in the nanomolar range. Charge transfer interactions with the FAD cofactor are the most accepted hypothesis, although the molecular mechanism is not completely understood [97]. Both COMFA studies and ALMOND procedure are involved in studying the structural activity relationship [83].

Studying MAO-A activity by using topological descriptors, pyrrole derivatives are one of the examples of 2D-QSAR. La Regina et. al. studied a series of new pyrrole derivatives that are synthesized and evaluated for their monoamine oxidase (MAO) A and B inhibitory activity and selectivity [98].

**Figure 4:**
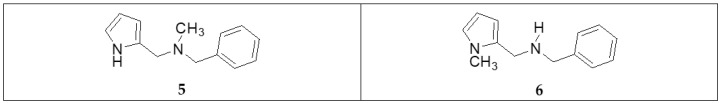
The structure of pyrrole derivatives; compounds **5** and **6** represent MAO-B and MAO-A selective inhibitors, respectively.

It was found that the most selective compounds were *N*-Methyl, *N*-(benzyl), *N*-(pyrrol-2-ylmethyl)amine (**5**) and *N*-(2- benzyl),*N*-(1-methylpyrrol-2-ylmethyl)amine (**6**) (Figure 4) for MAO-B [(1, SI) 0.0057] and MAO-A [(2, SI) 12500] inhibitors, respectively. Docking and molecular dynamics simulations play an important role in giving structural insights into the MAO-A and MAO-B selectivity. This could be explained by compound (**6**), as it forms a H-bond with Gln215 through its protonated amino group in the MAO-A binding site, while it is absent in the compound (**5**) MAO-A complex. Moreover, it could be noticed that **5** places its phenyl ring into an aromatic cage of the MAO-B receptor binding pocket, as it forms charge–transfer interactions. The slightly different binding pose of **6** into the MAO-B active site appears to be forced by a bulkier Tyr residue, which substitutes a smaller Ile residue present in MAO-A [99]. A study of 32 pyrrole derivatives (**2**) (Figure 3) and analogues with 28 topological descriptors was accomplished using SPSS software, through multiple linear regression [98]. A model with a squared correlation coefficient (0.9) was found [98]. Seven topological descriptors were chosen through stepwise regression to be in the last model: the total structure connectivity index (Xt), mean square distance index (MSD), all-path Wiener index (WAP), eccentric index (DECC), Kier flexibility index (PHI), superpendentic index (SPI) and the mean Wiener index (WA) [100], as well as the cross-validation strategy, were investigated. It is found that the positive coefficients of the indices DECC, MSD, PHI, and SPI confirm that an increase in their values produces higher values for the Ki. However, large WA, WAP, and Xt (negative coefficients) decrease the Ki values [101].

Altomare et al. [102] considered some parameters of some pyridazine derivatives (**3**) (Figure 3) in terms of their lipophilicity through measuring partition coefficients, thermodynamics, and physiochemical parameters of RP-HPLC retention. In a set of 14 pyridazine derivatives, using multiple linear regression (MLR), the equation yielded an r^2^ = 0.821 and q^2^ = 0.704 (cross-validation), confirming the importance of lipophilic, electronic, and steric properties in a way to explain the behavior of MAO-B inhibition [103]. The results showed that lipophilicity plays an important role in modulating MAO-B inhibition with no effect on A isoenzyme. Otherwise, electrostatic interactions and charge transfer bonding are critical factors in the interaction between inhibitors and the FAD cofactor of MAO-A. Altomare et al. concluded that most of the pyridazines derivatives showed selectivity towards MAO-B [102].

For phenylalkylamine scaffold (**4**), the structural properties for 29 compounds were analyzed by Norinder et al. and a set of physiochemical descriptors were calculated (Figure 3) [104]. Different partial least square (PLS) models were generated with squared correlation coefficients (r^2^) with a value greater than 0.85. The authors concluded that the most essential SAR and high in vivo and in vitro activities require (S)-stereochemistry and no substitution on the aliphatic chain. In order to develop the best QSAR, electronic descriptors are essential variables. In order to increase in vivo activities, it is essential to attach small, electron-withdrawing and hydrophilic substituents in ortho and meta positions. While symmetrical, electron-withdrawing and lipophilic substituents in the ortho position are important for in vitro activity [104]. The order of NHMe > NMe_2_ > NH_2_ > CHMe_2_ in para positions decreases both the in vivo and in vitro activity of the compounds [104].

## 7. Indole and Isatin Analogues

**Figure 5:**
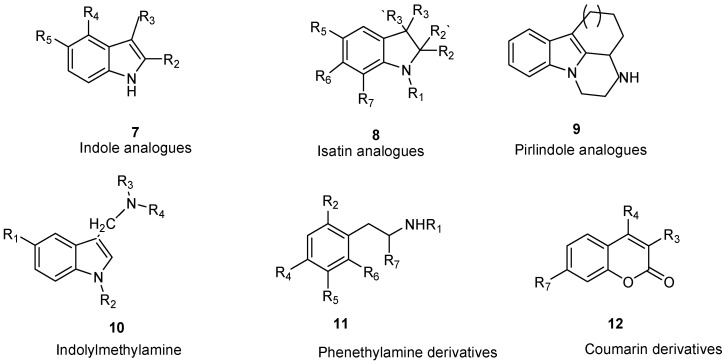
Main scaffolds of indole, isatin, pirlindole, indolylmethyl amine, phenethylamine, and coumarin studied as MAO-inhibitors. (R_1_ = OH, R_2 c_ = MeO, R_3_ = MeO, R_4_ = H or OH R_5_ = H, R_6_ = CHMe_2_, R_7_ = NHMe, NMe_2_, NH_2_).

Medvedev et al. studied a series of indole (**7**) and isatin analogues (**8**) as MAO-A and MAO-B inhibitors (Figure 5) [103]. It has been found that selective MAO-A or MAO-B inhibitors occur at different molecular sizes [103,105]. Using SYBYL software, COMFA analysis was used to study their QSAR model. As a previous step of molecular alignment, conformations with the lowest energy were calculated. In COMFA analysis, both electrostatic and steric fields were taken into account. PLS was used to determine the best formula relating the biological activity against different variables. Cross-validation r^2^ values were 0.743 and 0.603 for both MAO-A and MAO-B, respectively. In spite of common regions in MAO-A and MAO-B, the analysis also shows some different patterns in steric and electrostatic regions. These differences could help explain the distinct behavior of both enzymes in inhibitor selectivity [106,107].

## 8. Pirlindole Analogues

Medvedev et al. studied the inhibitory activity (IC_50_) of pirlindole analogues (**9**, Figure 5) with several substitutions at C8 by COMFA analysis [108]. The molecules were geometrically optimized and aligned by fitting the indole ring [108]. The molecular size analysis of the rigid pirlindole analogues (**9**, Figure 5) with (X, Y, Z; 13.0 × 7.0 × 4.4 Å) was more effective against the MAO-A enzyme receptor, even though the flexible analogues, regardless of size, showed acceptable potency against both MAOs [109,110].

## 9. Indolylmethylamine Derivatives

Maron et al. studied a set of indol ylmethylamines represented by structure **10** (Figure 5) [111]. Ki values were in the range of 0.8–>10^6^ nM and 0.75–476,000 nM for MAO-A and MAO-B, respectively. A semi-empirical method (AM1) was used for full geometry optimization. Superimposing the heavy atoms of the indole ring was accomplished by molecular alignment. SYBYL software with default parameters was used for COMFA analysis. Cross-validation squared correlation coefficients (q^2^) were 0.895 for MAO-A and 0.859 for MAO-B. In both enzymes’ models, similar contributions of steric, solvation, and electrostatic terms were found. Possible aromatic interactions between substitutions at C5 and Phe-208 of MAO-A and the possible hydrophobic van der Waals interaction between inhibitors and MAO-B (Ile-199) were inspected through computational simulations [111,112,113].

## 10. Phenethylamine Derivatives

In a series of 38 phenethylamine derivatives, COMFA analysis was developed to study the MAO inhibitory activity (IC_50_), represented by structure **11** (Figure 5). Different biogenic amine inhibitors can be generated from the same scaffold because this scaffold is found in many catecholamine neurotransmitters. The best COMFA model with r^2^ = 0.92 and q^2^ = 0.72 was gained for four components. The steric properties of the substituents played a more essential role than those of the electrostatic properties in this type of inhibitor. The molecular modeling of the crystal structure of clorgyline bound to MAO-A was performed to analyze the possible interactions with the backbone of the enzyme’s active site [110,111,112,113,114,115].

## 11. Coumarin Derivatives

Catto et al. studied a series of 3-, 4-, 7-polysubstituted coumarins (**12**) and their potential MAO inhibitory activity (Figure 5) [116]. The inhibitory potency was determined by testing the scaffold on rat brain mitochondria. Using SYBYL and CLIP software including steric, electrostatic, and lipophilic fields, different interactions were calculated. Generating optimal linear *PLS* estimations (GOLPE) analysis was carried out to extract the PLS coefficient. Both MAO activities were modulated using COMFA parameters including electrostatic, lipophilic, and steric fields. Ligand-based approaches provided major and valuable SAR information in the rational design of new MAO inhibitors. Ligand selectivity could be described through COMFA. Ligand-based methods can be joined with protein-structure models to identify the interactions involved in ligands and MAO enzyme binding domains. In order to study the isoenzyme selectivity, an additional 3D QSAR was developed that takes into account the difference between PIC_50_ in both MAOs. Enzyme selectivity mainly depends on the electrostatic field contrary to lipophilic and steric fields that were not major participants in enzyme selectivity. One of the most important factors affecting MAO selectivity was the different electron density localized on *α* and *β* positions of the bridge that links the coumarin core with a phenyl ring. Most of the molecular docking experiments were attempted to further study the interactions between coumarin derivatives and MAO enzymes [116,117,118].

## 12. MAO Inhibitors

MAO inhibitors differ in their origin; some are naturally available, while others are derived synthetically as shown in Table 1. Interestingly, MAO inhibitors show a different pattern of selectivity toward MAO-A and MAO-B with variant IC_50_, such as clorigyline with an IC_50_ value of 0.0049 µM toward MAO-A.

## 13. Pharmacophore Model Generation

**Figure 6:**
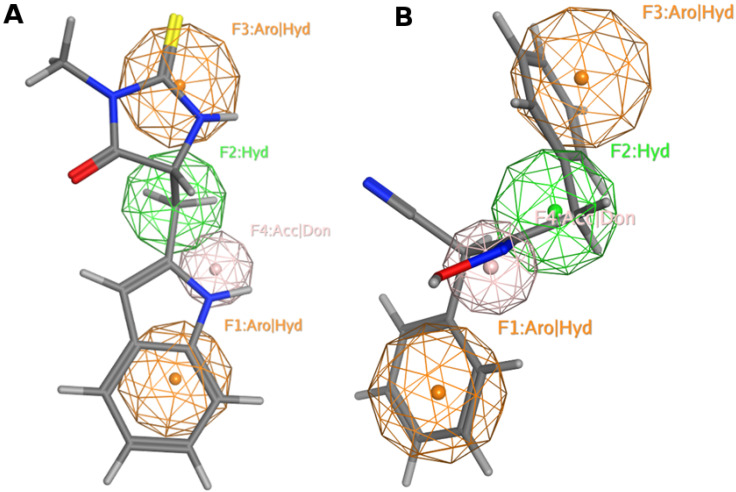
MAO-A pharmacophore model with (**A**) 5-((1H-indol-2-yl)methyl) -3-methyl- 2-thioxo imidazolidin -4-one and (**B**) NSC 38.

Pharmacophore model, a ligand-based drug design approach, represents ligands’ structural features that are recognized at the binding site to induce an activity. In this context, we generated MAO-A inhibitors’ pharmacophore model employing the coordinates of active reported inhibitors (Table 1) using MOE software [120]. MAO-A reported inhibitors were built, energy minimized, and superposed over clorigyline. The derived pharmacophore model recommends four functionalities illustrated as: F1 (Aro|Hyd); F2 (Hyd); F3 (Aro|Hyd); and F4 (Acc|don). Aro stands for aromatic ring, Hyd represents hydrophobic, Acc portrays H-bond acceptor, and don indicates H-bond donor. Our model shows that the MAO-A inhibitor should harbor two aromatic rings and one hydrophobic motif or three hydrophobic groups and one H-bond acceptor or donor moiety to invoke an activity (Figure 6A).

Next, we screened the pharmacophore model against the NCI database that contains 265,240 compounds [121]. Filtration of the NCI database was applied recruiting Lipinski’s rule [122] to retrieve drug-like molecules; 52,457 of the molecules were obtained and identified as hits (Figure 6B).

## 14. Docking

**Figure 7:**
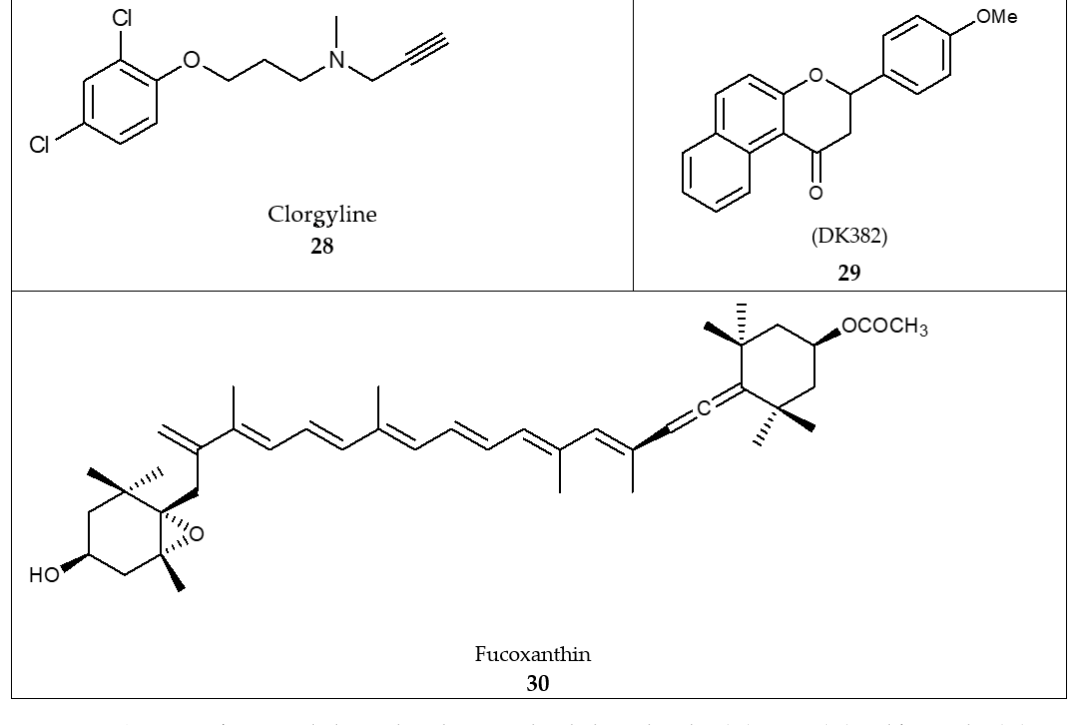
Structures of compounds designed employing in silico docking: clorgyline (**28**), DK382 (**29**), and fucoxanthin (**30**).

Reported docking studies [123] against MAO-A disclosed Tyr69, Asn181, Phe208, Val210, Gln215, Cys323, Ile325, Ile335, Leu337, Phe352, Tyr407, and Tyr444 as key binding residues. The results showed that the % inhibitory activity of 3-(4-methoxyphenyl)-2,3-dihydro-1H-benzo[f]chromen-1-one (DK382) was 83.8% (**29**, Figure 7) competitive to that of clorgyline (**28**, Figure 7) (80.8%). The MAO-A inhibitory activity of benzoflavanone (DK382) was comparable to that of clorgyline [123].

Other docking studies of coumarin derivatives and clorgyline against the MAO-A binding site revealed that aromatic (π-stacking) interaction guides ligand/complex interaction, particularly with Tyr407 [124]. The aromatic ring properly assists in the orientation of the ligand in the MAO-A catalytic domain [124]. Moreover, a reported pharmacophore model highlighted the significance of three hydrophobic features that accord with the aromatic interaction in the binding domain [124]. Further docking studies of fucoxanthin (**30**, Figure 7), a carotenoid in edible seaweeds, against MAO-A and B binding sites demonstrated that fucoxanthin accommodates the binding sites of MAO-A and B through hydrogen bonding and hydrophobic interactions [125]. Fucoxanthin exerted an inhibitory activity against MAO-A and B with IC_50_ values of 197.41 ± 2.2 and 211.12 ± 1.17 µM, respectively [125].

## 15. MAO-A Inhibitors as Anticancer Agents

MAO-A is a novel target gene of repressor element-1 silencing transcription factor (REST). It was reported that the neuroendocrine differentiation (NED) of prostate cancer (PCa) requires the downregulation of REST and activation of autophagy [126]. Studies showed that MAO-A inhibitors (pargyline and phenelzine) significantly reduced the NED and autophagy activation of PCa cells. Therefore, MAO-A inhibitors were considered a potential therapy for neuroendocrine tumors [126]. Yang et al. reported that a combination of isoniazid (INH), MAO-A inhibitor and tumor-targeting hepatomethine cyanine dyes proved to be a highly promising treatment tool for advanced PCa [127]. On the other hand, clorgyline, a selective irreversible inhibitor of MAO-A, prompted a mesenchymal to epithelial transition (EMT) in MDA-MB-231. Biological data showed that clorgyline induced E-Cardin (known epithelial protein marker) in breast cancer (MDA-MB-231) cells.

Moreover, clorgyline was shown to interfere with the β-catenin/p-GSK3β complex in addition to the E-cadherin/β-catenin complex. Overall, MAO-A is an essential regulator of EMT in breast cancer. Contrastingly, clorgyline was shown to reduce temozolomide (TMZ)-resistant glioma progression. Clorgyline induced cytotoxicity and reduced tumor cell invasion [39]. Thus, MAO-A inhibitor either alone or in combination with a low dose of TMZ may be potential therapy for the treatment of brain tumors [39].

## 16. Conclusions and Perspectives

MAO-A and MAO-B are highly expressed in diverse human cancers. MAO-A is expressed in prostate and lung cancer, whereas MAO-B is expressed in gliomas and renal cancer. The increased production of ROS mediated by MAO-A oxidative deamination activity might aggravate tumorigenesis and metastasis in high-grade tumors. The chemical inhibition of MAO-A might present a valuable therapeutic approach for cancer treatment. In this review, we reported different approaches exploited in the design and development of selective MAO-A inhibitors accompanied by biological activities. Additionally, we applied a ligand-based drug design approach to generate a pharmacophore model for active MAO-A inhibitors. Our pharmacophore model suggests that the MAO-A inhibitor should harbor two aromatic rings and one hydrophobic motif or three hydrophobic groups and one H-bond acceptor or donor moiety to elicit an activity.

## Figures and Tables

**Table 1:** The chemical structures of natural and synthetic MAO inhibitors with their IC_50_ values. NA stands for not available.

Scaffold	Selectivity	IC_50_ MAO-A	IC_50_ MAO-B	Chemical Structure
Chalcones [119]	Nonselective	43.4 µM	43.9 µM	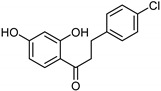 Xanthoangelol **13**
Flavonoids [119]	Selective	1.23 µM	NA	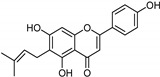 **14**
Coumarins [119]	Nonselective	8.9 nM	8.9 nM	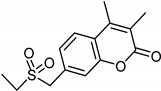 **15**
Xanthones [119]	Nonselective	13.92 µM	13.92 µM	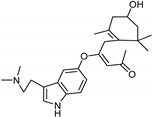 Desmodeleganine **16**
Nicotinamide [119]	Selective	0.045 µM	26 µM	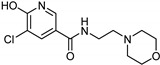 **17**
Caffeine [119]	Selective	34 µM	0.148 µM	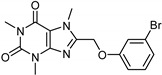 **18**
Indole alkaloids [119]	Selective	0.07 µM	NA	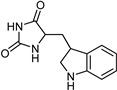 **19**
Anthraquinone [119]	Selective	2.5 µM	NA	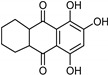 Purpurin **20**
Synthetic [95]	Selective	5.5 nM	150 nM	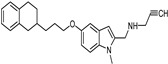 **21**
Synthetic [96]	Selective	0.49 µM	NA	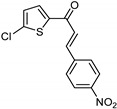 **22**
Synthetic [96]	Selective	0.14 mM	NA	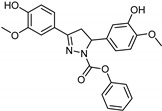 **23**
Synthetic [96]	Selective	0.06 µM	NA	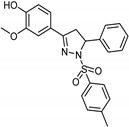 **24**
Synthetic [96]	Selective	0.01 µM	2.15	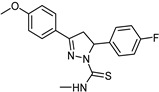 **25**
Synthetic [96]	Selective	NA	20 nm	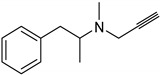 Selegiline **26**
Natural [96]	Selective	NA	8.3 μM	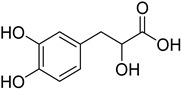 Danshensu **27**

## Sample Availability

Samples of the compounds are available from the authors.

## Abbreviations

MAO	Monoamine Oxidase
ROS	Reactive Oxygen Species
FAD	Flavin Adenine Dinucleotide
PCa	Aggressive prostate cancer
NSCLC	None-small cell lung carcinoma
EMT	Epithelial to Mesenchymal Transition
QSAR	Quantitative structure–activity relationship
CoMFA	Comparative molecular field analysis
PLS	Partial least square
REST	Repressor element-1 silencing transcription factor
NED	Neuroendocrine differentiation
PCa	Prostate cancer
